# Cancer-Nano-Interaction: From Cellular Uptake to Mechanobiological Responses

**DOI:** 10.3390/ijms22179587

**Published:** 2021-09-03

**Authors:** Ahmad Sohrabi Kashani, Muthukumaran Packirisamy

**Affiliations:** Optical Bio-Microsystem Lab, Micro-Nano-Bio-Integration Centre, Department of Mechanical, Industrial and Aerospace Engineering, Concordia University, 1455 De Maisonneuve Blvd. W., Montreal, QC H3G 1M8, Canada; ah_sohra@encs.concordia.ca

**Keywords:** nano-bio-interaction, nanoparticle, mechanobiological properties, cancer cells, cell mechanics, migratory index

## Abstract

With the advancement of nanotechnology, the nano-bio-interaction field has emerged. It is essential to enhance our understanding of nano-bio-interaction in different aspects to design nanomedicines and improve their efficacy for therapeutic and diagnostic applications. Many researchers have extensively studied the toxicological responses of cancer cells to nano-bio-interaction, while their mechanobiological responses have been less investigated. The mechanobiological properties of cells such as elasticity and adhesion play vital roles in cellular functions and cancer progression. Many studies have noticed the impacts of cellular uptake on the structural organization of cells and, in return, the mechanobiology of human cells. Mechanobiological changes induced by the interactions of nanomaterials and cells could alter cellular functions and influence cancer progression. Hence, in addition to biological responses, the possible mechanobiological responses of treated cells should be monitored as a standard methodology to evaluate the efficiency of nanomedicines. Studying the cancer-nano-interaction in the context of cell mechanics takes our knowledge one step closer to designing safe and intelligent nanomedicines. In this review, we briefly discuss how the characteristic properties of nanoparticles influence cellular uptake. Then, we provide insight into the mechanobiological responses that may occur during the nano-bio-interactions, and finally, the important measurement techniques for the mechanobiological characterizations of cells are summarized and compared. Understanding the unknown mechanobiological responses to nano-bio-interaction will help with developing the application of nanoparticles to modulate cell mechanics for controlling cancer progression.

## 1. Introduction to Cancer-Nano-Interactions

Cancer is one of the leading causes of death in the world [1], and over the past decades, intensive efforts have been made to develop new and effective methods for the diagnosis and treatment of cancer [2,3,4,5]. Surgery, radiation, and chemotherapy are the main classical techniques for cancer treatment [6,7]. However, due to their limitations and the undesirable side effects such as systemic toxicity, lack of precise targeting, and non-specific distribution, their application in cancer treatment is not satisfactory [8]. Developing novel therapeutic approaches to overcome these limitations and deliver agents only to the tumor sites without inducing negative effects on healthy tissues or organs is an important challenge in cancer treatment [9]. In recent years, with progressing nanotechnology techniques, the concept of the targeted therapy and controlled releasing have received great attention in cancer treatment. In the targeted therapy, nanoparticles (NPs) with specific properties, nanomedicine, are designed to specifically transport therapeutic agents to tumor sites and to release under controlled conditions [10]. This strategy could potentially overcome the limitations of conventional methods and improve the cancer treatment outcomes by distinguishing malignant cells from non-malignant cells and selectively kill malignant cells [11,12,13]. 

Bio-distribution, biocompatibility, biodegradability, and systemic clearance are the general challenges of using NPs in the targeted therapy [14]. An effective NP-based drug delivery system should predict and control the fate of NPs in the biological environment [15]. To develop and achieve a sound and efficient NPs-based system, we need to enhance our understanding of the nano-bio-interaction (NBI) happening between nanomaterials and a complex heterogeneous biological environment [16]. At the cellular level, the NBI occurs at the interface of NPs surface and cell membrane. The interaction behavior of NPs is highly dependent on the physical and chemical properties of NPs. Therefore, it is crucial to obtain a complete understanding of NPs cellular uptake, nanotoxicity, and intracellular distribution with respect to their properties to design safe NPs and control targeting [17,18]. 

**Figure 1:**
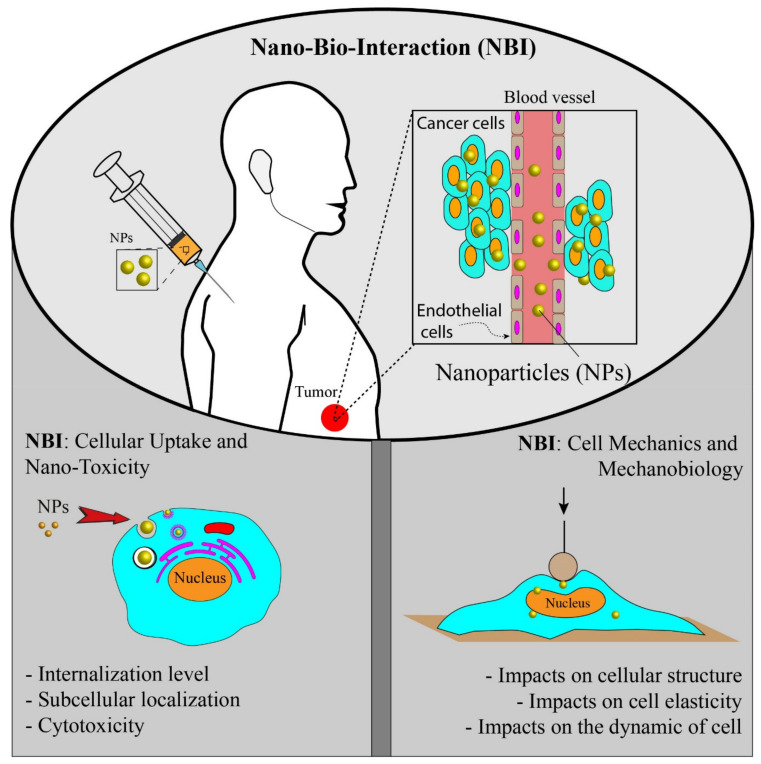
Nano-bio-interaction from two different aspects: (1) study of the cellular uptake and toxicity, (2) study of the mechanics and mechanobiology of cells due to the cellular uptake of NPs.

In the NBI field, researchers have long studied physiochemical properties of NPs and their effects on cellular uptake, cytotoxicity, and intracellular fate [19,20], while few researchers have addressed NPs’ exposure effects on cell mechanics and biological activities (Figure 1). The mechanobiological properties such as stiffness play an important role in modulating the cellular behavior, and any abnormal changes could cause cellular dysfunctions such as impaired migration, impaired differentiation, and impaired wound healing [21,22,23,24,25]. For example, during cancer progression, the stiffness of cells is reduced, giving a high ability of migration and invasion [21,25]. Mechanobiological measurements provide valuable information on cell functionality and health [26]. The cellular uptake of NPs and their direct or indirect interactions with intracellular compartments could disorganize the cytoskeletal structures and consequently alter the cell mechanics and cellular functions [16]. Hence, mechanobiological measurements should be suggested to study NBI and explore how NPs modulate the mechanobiological properties of cells. In addition to biological responses, mechanobiological measurements could potentially be used both to evaluate the effectiveness of NP-based systems and manipulate NBI for therapeutic applications. 

In this review, both the above-mentioned aspects of NBI are discussed (Figure 1), but the main focus is given to the mechanobiological responses of cells and how they could be used to assess NBI. Despite many advances in NBI, the impact of NPs on mechanobiological changes still remains poorly understood. Mechanobiological responses could provide additional information on NBI and allow designing more effective nanomedicines for therapeutic applications. Here, we briefly review important NPs’ physiochemical properties and their effects on NBI, then the interactions between nanomaterials and cells are discussed in the context of cell mechanics, and important tools for mechanobiological measurements are discussed. 

## 2. Nano-Bio-Interaction: Cellular Uptake and Toxicity

A nanoparticle is defined as a particle that is 1–100 nanometres in one of its dimensions [27]. NPs possess various shapes, such as spheres, rode, wires, stars, planes, etc. They possess unique properties including thermal, optical, magnetic, electrical, and mechanical properties, high surface energy, and a high surface-to-volume ratio that is not found in their bulk counterparts. These properties make them suitable for application in biology and medicine [28]. Nanomedicine is one of the main applications of NPs in medicine. NPs could carry therapeutic agents and other biological materials and deliver them to diseased sites in body. To design nanomedicines for cancer therapy, we need to efficiently deliver NPs to tumor sites in the body with cellular and oftentimes subcellular precision. In order to provide an effective NP-based drug delivery system, it is essential to have full control over NPs cellular uptake, NPs internalization level, NPs localizations, and NPs distributions within cells. NBI is a complex, dynamic, and multi-parametric phenomenon [29]. Hence, before using NPs for therapeutic application, we need to enhance our fundamental understanding of how NPs’ physicochemical properties affect their interactions with biological systems. Biocompatibility and toxicity are two important parameters that are used to assess NPs for therapeutic purposes. Biocompatibility shows the ability of NPs to provide the designed and desired functions in terms of cancer therapy without inducing undesirable local and systematic effects in the recipient [30,31]. In the same context, toxicity is defined as the ability of NPs to affect the normal physiology of cells adversely and directly disrupt the structure of cells or tissue [31,32,33]. Several investigations have shown the importance of NPs properties in biocompatibility and toxicity [19,34,35,36,37]. Apart from the physicochemical properties of NPs, other factors or conditions such as cell lines [38], cell size [39], cell sex [40,41], incubation time [37], NPs concentration [37], protein absorption [42], and evaluating methods may affect the biocompatibility and toxicity of NPs. In the following parts, briefly, the main physicochemical properties of NPs impacting the quality of NBI in terms of cellular uptake, internalization, toxicity potential, and biocompatibility are reviewed.

### 2.1. Effects of NPs’ Size

**Figure 2:**
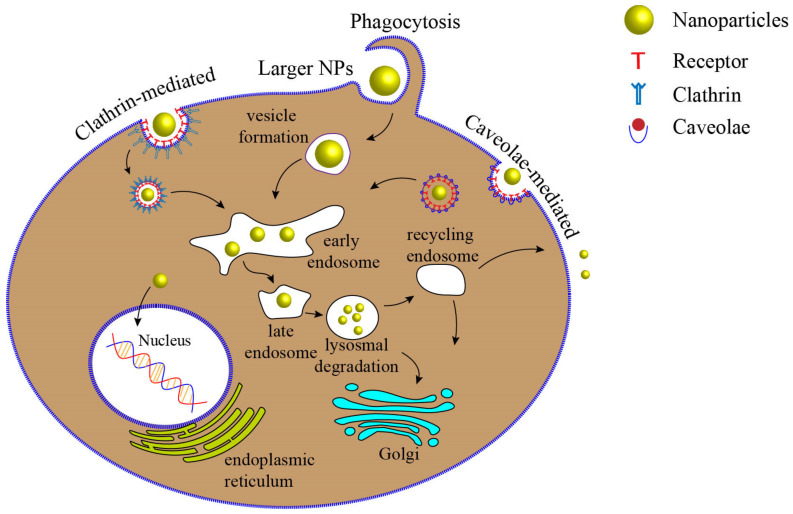
Different endocytosis mechanisms by which NPs can enter cells. Larger particles can be taken up by phagocytosis, while smaller particles can bind to some proteins such as Clathrin and Caveolae on the membrane of cells and enter cells. NPs are engulfed with the cell membrane and entrapped in the cellular vesicle. Then, the vesicles are uncoated and delivered to specialized intracellular components. Early endosomes fuse vesicles and transport particles to different destinations. The early endosome next matures down to the late endosome, and they fuse with lysosomes [17,43].

The cellular uptake of NPs strongly depends on the NPs’ size. With the aid of the endocytosis process (Figure 2), cells can uptake biomolecules, and due to the similar size of NPs to biomolecules, NPs could enter into cells through the same mechanism. NPs can be taken up by cells, either by engaging with some proteins on the membrane of cells such as Clathrin or Caveolae or by other mechanisms such as pinocytosis and phagocytosis [17,43,44,45]. It has been shown that each type of particle may prefer a different pathway for cellular internalization. NPs, depending on their size, may choose different endocytic mechanisms to enter cells [17,46,47]. Larger solid particles generally enter cells through phagocytosis with good efficiency. This process might take thirty minutes to several hours with respect to cell type and NPs properties. Larger particles could even enter the nucleus; for example, larger gold NPs have been found to be able to localize in the nucleus of HeLa cells during cell division [48,49]. Particles in the size range of 200–1500 nm in diameter can be taken up by phagocytosis [17]. Smaller particles (less than 100 nm) adhere to Clathrin and Caveolae proteins and form endocytosis, helping cells engulf particles [50]. The ability of NPs to enter the cells may be increased by reducing their sizes [51]. However, smaller NPs, due to their low binding tendency to receptors, have less chance to be engulfed by the membrane, so they need to form clusters on the cell membrane [52]. Several investigations have shown that 50 nm is the optimal size for the highly efficient cellular uptake of gold NPs [53,54,55]. Ko et al. [56] reported that spherical NPs in the 30–50 nm range have higher internalization efficiency to human adipose-derived stem cells (ADSCs) than that of NPs with sizes of 15, 75, and 100 nm. NPs aggregation could also influence cellular uptake and interactions with cells membrane. Albanese et al. [53] observed a 25% decrease in the uptake of aggregated NPs with HeLa and A549 cells compared to monodisperse NPs. Moreover, as the size of NPs plays a significant role in the endocytosis efficiency, the cytotoxicity of NPs may be influenced by particle size. Some studies have shown that gold NPs with smaller sizes are more toxic to the cells compared to larger NPs [46]. Smaller NPs have a higher surface area to volume ratio, enabling them to interact more effectively with cellular and subcellular compartments. On the other hand, smaller particles have a better chance to penetrate intracellular locations such as mitochondria and nucleus, making them more toxic [57,58]. For example, Pan et al. [46] showed that smaller gold NPs (1.4 nm) induce a higher cellular toxicity compared to larger NPs (15 nm) in HeLa and L929 cells. While NPs size is a determining factor in designing nanomedicines or targeting specific subcellular localizations, their interaction with biological environments before reaching the target site may influence the way they enter cells and thus affect their toxicity [59,60].

### 2.2. Effects of NPs Shape

In addition to the size of NPs, their shapes may influence NPs’ internalization ability and NPs toxicity potential. Due to their shape, NPs may interact differently with cellular and subcellular compartments of cells. Spherical NPs have shown a higher level of internalization compared to their non-spherical counterparts [20,61]. While nanospheres are good candidates for drug delivery, anisotropic nanostructure could provide better efficiency due to their higher surface/volume ratios and carry more drug concentration to the desired sites [14]. NPs with different shapes exhibit different abilities to enter cells because of the different contact areas with the cells membrane. For example, nanorods exhibit lower internalization ability than spherical particles and need a longer time for membrane wrapping [52]. However, by reducing the aspect ratio of nanorods (length to width of particles), their cellular uptake can be significantly enhanced. The findings of Chithrani et al. [52] revealed that sphere-shaped gold NPs with the size of 14 nm and 74 nm are taken up 5 and 3.75 times more than 74 × 14 (nm) rod-shaped gold NPs, respectively, by human breast cancer cells (MCF-7). Xie et al. [62] studied the effects of gold NPs morphology on their internalization ability. They considered star-, rod-, and triangle-shaped nanogold with similar sizes coated with methoxy polyethylene glycol to investigate their internalization level to mouse leukemic monocyte macrophage. They found that the triangle-shaped NPs tend to enter the cells with higher efficiency than other shapes, and gold nanostars displayed the lowest ability. Furthermore, they observed that each type uses a different endocytosis process to penetrate cells membrane, highlighting the role of NPs geometry in modulating the intracellular fate. In another work, Herd et al. [63] found that spherical NPs prefer to penetrate cells through Clathrin-mediated endocytosis, while worm-like NPs undergo phagocytosis. These data suggest the important role of morphology in nanomedicines designs and targeting specific subcellular sites. 

### 2.3. Effects of NPs Surface Charge and Coating

NPs’ surface charge impacts the electrostatic interactions between cell membranes and NPs; hence, it plays a determining factor in cellular uptake. It has been shown that both sides of a bilayer phospholipid membrane of cells are negatively charged, and the cell membrane has a hydrophobic surface [47,64]. Theoretically, the importance of surface charge has been studied on the interactions of NPs and cell membranes. It was shown that cationic charged NPs have better thermodynamical interaction with phospholipid membranes [65,66]. In contrast to anionic and neutral NPs, the positively charged NPs adhere readily to the cell membrane and enhance the membrane-engulfing process [17,47]. Negatively charged NPs induce local disorders in their local contact with cell membranes, making their interaction unfavorable. Cho et al. [67] found that the uptake of cationic gold NPs is five-fold greater than that of their anionic counterparts. Their findings revealed that gold NPs could even directly diffuse to cells through generating holes in the cell membrane, whereas negatively charged and neutral gold NPs are internalized only through endocytosis. In another study, Arvizo et al. [68] reported that cationic gold NPs depolarize the cell membrane and increase the concentration of Ca^2+^ within cells. The depolarization might reduce the proliferation and viability of normal cells via changing the intracellular pathways. Hauck et al. [69] studied the uptake of very negative and very positive gold nanorods into HeLa cells at different concentrations. They showed that maximum and minimum uptake take place for positive and negative, respectively. Jiang et al. [34] demonstrated that the surface charge might modulate NPs’ size-dependent uptake into cells. They observed that with anionic gold NPs, cellular uptake is decreased as their size increases, whereas, for cationic gold NPs, the level of internalization is enhanced by decreasing their sizes. 

Understanding the role of surface coating on the internalization ability and the intracellular pathways is needed to design efficient nanomedicine. It has been shown that the surface functionalization significantly influences the intracellular pathways, so we could dictate NPs to interact differently with biological systems by controlling the surface coating. In order to control the NPs delivery to a specific site for therapeutic purposes, the surface of the NPs is functionalized with biomolecules such as peptide ligands, antibodies, or various chemical groups [34,70]. These ligands can guide NPs to the intended sites by recognizing specific receptors on the surface of cells, and then, NPs can be taken up via receptor-mediated endocytosis. In addition, the surface coating can be used to improve the stability and biocompatibility of NPs. For example, the findings of Bartczak et al. [71] showed that with diacetylene-containing ligand, the stability of gold NPs could be remarkably increased under different pH and temperatures. Surface functionality also could impact cytotoxicity. For instance, Chompoosor et al. [72] studied the toxicity of NPs in HeLa cells with respect to the hydrophobicity and found that by increasing the hydrophobicity of particles, the cytotoxicity increased. 

## 3. Nano-Bio-Interactions: Cell Mechanics and Mechanobiology

NBI behaviors can be evaluated from a mechanics point of view. The presence of NPs within cells might directly or indirectly impact the function of cells, and their effects can be reflected in the mechanics or mechanobiology of cells. Mechanobiology is an emerging multidisciplinary field at the crossroads of biology, bioengineering, and biophysics. Mechanobiology describes how cell mechanics and mechanical forces influence cell behavior, cell morphogenesis, and diseases such as cancer [23,73]. In the context of mechanobiology, the mechanical properties of cells play significant roles in sensing and responding to their external surrounding [25,73,74,75]. With the help of the cytoskeleton, cells can resist the deformation induced by their microenvironment and alter their shapes during movement by polymerizing or fluidizing the polymeric structure of the cytoskeleton [76,77,78]. During this process, the structural stiffness of the cells is either reinforced or reduced, helping cells to maintain their physiological processes and continue their biological functions. 

The cytoskeleton structure (cell mechanics) plays a central role in controlling cellular functions, and any abnormal changes may cause dysfunctions. For example, during cancer, the elasticity (stiffness) of cells is decreased, enabling them to invade other organs and metastasize [25,79]. In NBI, where NPs interact with cellular organelles and cytoskeletal structures, they might influence cell mechanobiology and, in return, alter cellular responses such as cell migration, cell adhesion, and cancer metastasis. In the following sections, briefly, the biomechanics of cells and measurement techniques are reviewed, and the effects of NPs on the cytoskeleton organization, cell migration, and cell stiffness are then discussed. 

### 3.1. Basic Components of Cells and Biomechanics

**Figure 3:**
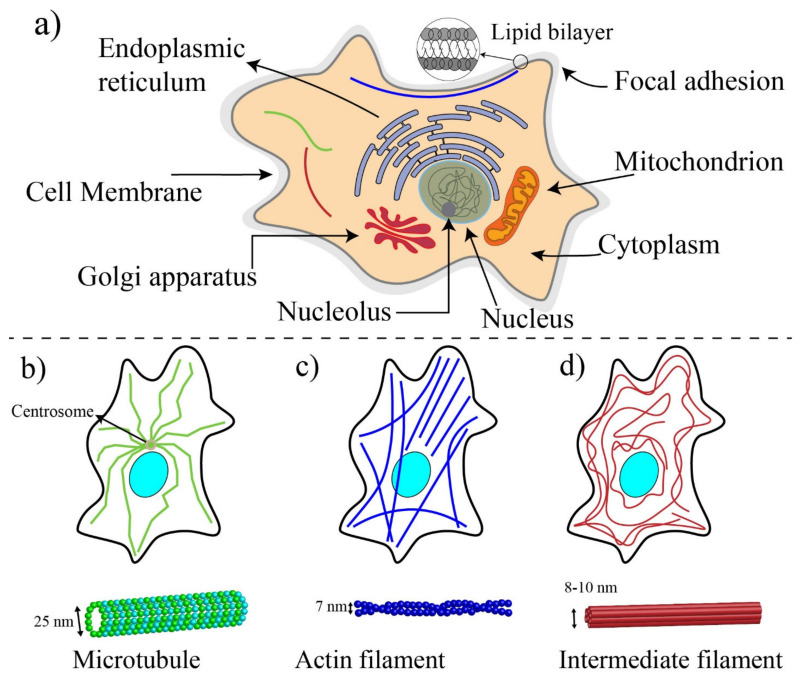
(**a**) Schematic showing typical eukaryote cells, (**b**) microtubules (they are in curved format), (**c**) actin filaments or long stress fibers (they are in linear format), and (**d**) intermediate filament (they are extending from the nucleus to the periphery of cells).

Cells are the basic functional unit of living organisms. Unlike plant cells (prokaryotic), animal cells (eukaryotic) do not have enclosing cell walls, and they are surrounded only by cell membranes [80] (Figure 3a). The cellular membrane is a thin (5–10 nm thickness) and permeable lipid bilayer that controls the flow and movements of ions and molecules between the interior of cells (cytosol) and the extracellular environment [76]. To retain the structural integrity of cells, a specialized cellular structure is required. This cellular structure, the cytoskeleton, determines the mechanobiological properties of cells (such as stiffness) and influences the shape, division, and functions of cells [81,82]. In addition to cytoskeletal proteins, other cellular components such as the membrane, the nucleus, and the cytoplasm could impact the mechanic of cells to some extent [21]. 

A schematic drawing of a eukaryotic cell is illustrated in Figure 3a. The nucleus, the mitochondria, endoplasmic reticulum, Golgi apparatus, and cytoskeleton are the main components of the internal part of a typical eukaryotic cell [83]. The nucleus of the cell is the largest organelle among subcellular components [84] and is located within the central region of cells and includes two regions: the internal region containing DNA and proteins and the outer boundary of the nucleus or karyotheca, which is a lipid bilayer similar to the membrane of cells. Regulating the gene expression is the main role of the nucleus, and to some extent, contributes to the cell mechanics [85]. The cytoplasm of eukaryotic cells includes all the material within the cell and outside the nucleus, such as proteins, protein complexes, and organelles [86]. Cells are dynamic living systems, and their mechanobiological properties allow them to sense microenvironmental changes and convert stimuli and changes into biological signals [21,87]. 

The cytoskeleton is made with a complex network of protein fibers and biopolymers embedded in the cytoplasm. In addition to maintaining the integrity of cells, the cytoskeleton provides pathways for molecular motor proteins to shuttle cargo between different regions of cells and generate and transmit cellular forces [81]. In response to the mechanical changes in their microenvironments, cells can either reinforce their cytoskeleton by polymerizing their structural proteins or fluidize their cytoskeleton to reduce their stiffness. Microtubule (MT), intermediate filament, and actin filament (F-actin) are three major fiber parts of the cytoskeleton [88] (see Figure 3b–d). MTs (diameter ≈25 nm), composed of two subunits (α and β tubulins), are stiff and hollow structures of the cytoskeleton, radiating outward from the central organelle. Intermediate filaments provide the strength, integrity, and organization of both the cell and nucleus. The intermediate filaments (diameter ≈10 nm) are composed of various proteins known as protofilaments (protein lamin, vimentin, keratin). These proteins are bundled around each other in a rope-like structure to form the final intermediate filaments. Intermediate filaments have a Young’s modulus between 1 and 5 GPa, and their length is between 1 and 3 μm. Intermediate filaments within the cytoplasm act as “stress absorbers” and organizes the position of organelles in cells [89].

Actin filaments are the main structural component of cytoskeleton, and with the help of non-muscle myosin II proteins, they provide the required forces for the movement and contraction of cells [90,91]. The actin filaments are composed of two different actin chains: F-actin and G-actin, which are twisted around each other. G-actin monomers are polymerized to form stiff F-actin with a modulus elasticity between 1 and 2 GPa [76,92]. The dimeter of F-actin varies from 5 to 9 nm and has a length in the order of ten micrometers. F-actin filaments are linked to each other during cell migration to form branches at a 70-degree angle from the original filament, enabling the cell membrane to protrude outward [76]. With the aid of non-muscle myosin II, two or more F-actin filaments are bundled in parallel to provide stress fibers. Myosin II is a molecular motor protein that makes F-actin filaments slide past each other to generate forces within cells [90]. Myosins directly impact cell mechanics, elasticity, cells adhesion, and mechanosensing [93]. The force generated by myosin is transmitted through focal adhesions, aggregates of cytoplasmic proteins at the inner surface of the membrane, to the interface of the integrin and extracellular matrix, and these forces are considered as traction forces to help cells move forward during cell migration [76,89].

Among these three different components of the cytoskeleton, actin filament plays the most important role in the structural integrity and deformability of the cell. Intermediate filaments are also able to tolerate some reasonable extent of deformations by engaging in shear stress. MTs play an important role in the cytoskeleton stability but contribute less to the mechanical integrity than the two other filaments [81,94,95].

### 3.2. Techniques for Mechanobiological Characterizations

**Figure 4:**
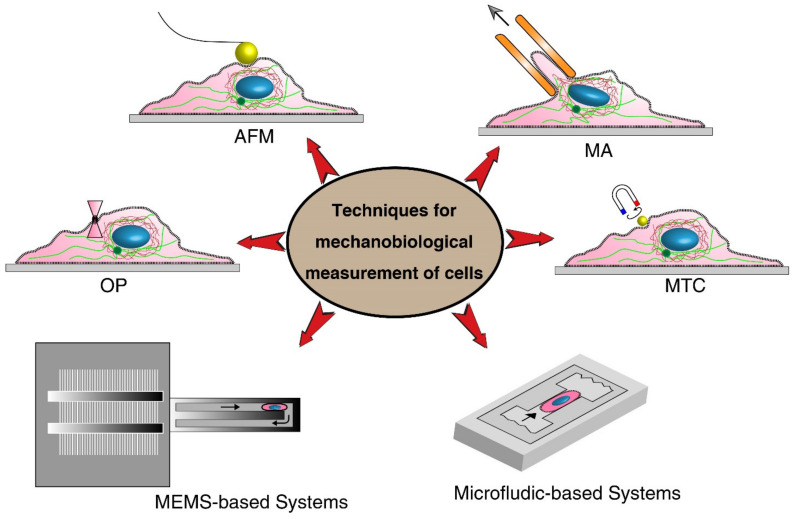
Different tools for the mechanical characterizations of living cells, Atomic Force Microscopy (AFM), optical tweezer (OP), micropipette aspiration (MA), magnetic twisting cytometry (MTC), MEMS, and microfluidic techniques.

Various techniques can be implemented to measure the mechanobiological properties of single cells, such as viscosity and elasticity. The elastic modulus and viscosity modulus are typically used to express the mechanical properties of cells [25,88]. In the elastic modulus, the applied forces are related to cell deformation, while in the viscosity, time-dependent stress relaxation is measured in response to a step displacement [21,96]. Sufficient and controlled forces need to be applied to the cells to measure their mechanical properties. Based on the types of forces, different microrheological tools have been developed to measure mechanical properties. The most used methods for experimental measurements are shown in Figure 4. Classical methods such as Atomic Force Microscopy (AFM) [97], micropipette aspiration (MA) [98], optical tweezer (OP) [99], and magnetic twisting cytometry (MTC) [100] are preferred because of their high-resolution measurements. However, they are tedious, and the measurements take a long time. With MEMS (micro-electromechanical systems) [101] and microfluidic devices [102], mechanobiological properties can be measured at a higher speed, but their resolution is not as high as that of classical methods, and most of them are able only to measure deformability-related parameters, not elastic and viscosity modulus [103]. To enhance the accuracy of these methods, in parallel to experimental measurements, computational analyses need to be carried out; however, they may impose a level of complexity [76]. Table 1 shows the limitation and advantages of different techniques. The technique can be chosen based on the type of cells and depending on the specific desired information. 

#### 3.2.1. Classical Methods

Classical methods provide a high-resolution measurement on the mechanobiology of single cells; however, they suffer from tedious, low-throughput, and long-processing measurements. Among various measuring techniques, AFM has been extensively used to study the mechanobiological properties of nano-treated single cells. This section will mostly focus on the AFM technique and briefly discuss other main classical methods.

Optical tweezer is one of the popular classic techniques for the manipulation and mechanical characterizations of suspended cells. In this technique, a focusing laser beam, introduced from a high numerical aperture objective, is utilized to trap single cells close to the beam focus. OP could apply time-varying stretching forces ranging from 0.1 to 100 pN onto the trapped cells to characterize mechanical properties. The mechanical properties of cells can be quantified by calibration techniques. Even though OP is an effective technique for mechanobiological measurements, it may induce unwanted detrimental effects to cells due to using a high-powered laser, altering the mechanics of cells [88,107].

Magnetic twisting cytometry is another well-established method for the mechanobiological characterization of living cells. In this technique, magnetic beads attached to the cells impose a quantified external force on the portion of cells under an external magnetic field. The magnetic-field-induced bead displacement is tracked to characterize the viscoelastic properties of cells. The applied stress can be controlled by translocating and regulating the external field. MTC offers various advantages over other methods. MTC generates both liner force and twisting torque, magnetic manipulation does not cause light-induced damage as in optical trapping, and MTC allows parallel simultaneous measurements. There are also some disadvantages associated with this method. It is not easy to control the region where beads are bound to cells, and more importantly, beads lose magnetization with time and need to be re-magnetized to maintain the torque applied [106,116].

Micropipette aspiration is a traditional method used to deform cells by imposing gentle suction to a micropipette. MP deforms individual cells in whole, and their deformations are measured to quantify the mechanobiological properties. This technique applies a small negative pressure into the glass micropipette with an inner diameter smaller than cells, causing them to deform and elongates a portion of them into the pipette. Several parameters such as the suction pressure, the diameter of pipette orifice, and the protrusion length of cells in the pipette are measured to derive the mechanobiological properties (stiffness) of aspirated cells. This technique has been used to measure the mechanical properties of numerous types of cells such as HeLa [117] and human leukocytes cells. Although MA offers a straightforward and well-established method for the mechanical characterization of individual cells, requiring special equipment and involving delicate procedures are the main challenges [75,118,119].

**Figure 5:**
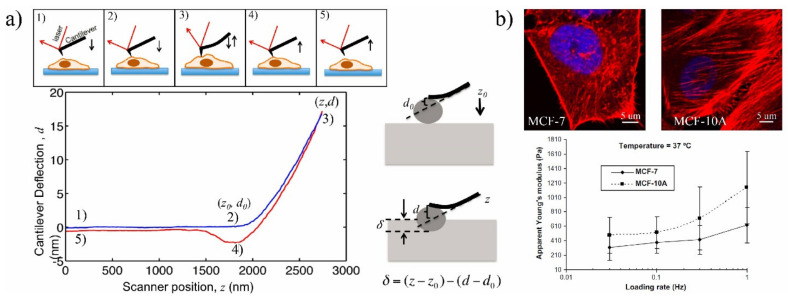
(**a**) AFM indentation and interpretation of the curve: (**1**) AFM above the cell surface, (**2**) AFM in contact with the cell surface, (**3**) motion of the AFM cantilever to contact the cell surface and indent into cell until the setpoint, (**4**) AFM tip detaching from the sample (AFM tip-cell adhesion), (**5**) returning to initial position. Reprinted with permission from [128]. Copyright Journal of Visualized Experiments 2013. (**b**) Cell elasticity measurement of human breast cancer cells with AFM and visualization of actin filaments in both cell lines. Reprinted with permission from [104]. Copyright Elsevier 2008.

Over the past three decades, AFM has been used as a key tool for simultaneous morphological and mechanobiological characterizations of different living cells, such as human kidney cells [120], human bladder cancer [121], ovarian cancer cells [122], and breast cancer cells [123]. The AFM method was introduced in 1986 to imaging and manipulating matter at molecular and cellular scales [124]. AFM can be used in liquid environments, and it has a flexible cantilever (several micrometers) at the end to probe the sample topography and measure forces between the tip and sample with piconewton sensitivity. The AFM technique is not a high-throughput method, but it has a simple principle of operation, allowing users to adjust this technique to measure the desired mechanobiological property. However, it has several intricacies that make the acquisition of quantitative data complex. To apply deformations, the AFM tip is vertically indented into the cell until the pre-set loading force, and the applied force, which is proportional to the cantilever deflection, is recorded (Figure 5). The motion of the cantilever can be measured optically by a beam of laser or through sensing elements built into the cantilever itself. Then, the AFM tip is controlled to return to its original position. During the approach–retract process, the cantilever deflection versus the vertical displacement of the AFM probe is recorded. The approach curve along theoretical models can be used to extract the cellular Young’s modulus, while the retract curve can be used to quantify the adhesion force [97,125,126,127].

There are different contact models to extract the mechanical properties from the AFM-obtained curve. The most commonly used models for estimating the cellular Young’s modulus include Hertz, Sneddon, Johnson–Kendall–Roberts (JKR), Derjaguin–Muller–Toporov (DMT), and Oliver–Pharr [23]. Each model can be used based on the different AFM tip geometries and sample properties. The Hertz is the most frequently used model to approximate the contact between the AFM tip and the sample. Three assumptions are considered for using the Hertz model: the AFM tip is a perfect sphere, linear strain–stress relationship (maximum 30% indentation of sample thickness), and the sample deformation is fully reversible. If these conditions are met, the Hertz model could extract mechanobiological properties by defining the contact point, which is difficult to determine, particularly for mammalian cells with complex surface morphologies [23,97]. With AFM, forces as small as 10^-11^ N can be measured.

#### 3.2.2. MEMS- and Microfluidic-Based Techniques

Although classical methods provide high-resolution measurements, single-cell analysis with classical methods is a very time-consuming process. MEMS-based approaches, including microfluidic techniques [129], could provide high-throughput alternatives that can clinically be used for the deformability characterization of individual cells [130]. Microfluidic-based systems could characterize the mechanobiological properties of thousands of cells in a short time. Their resolutions might not be competitive with classical tools, so they mostly focus on deformability-related parameters rather than elastic properties. In the following, a few prominent techniques are discussed.

**Figure 6:**
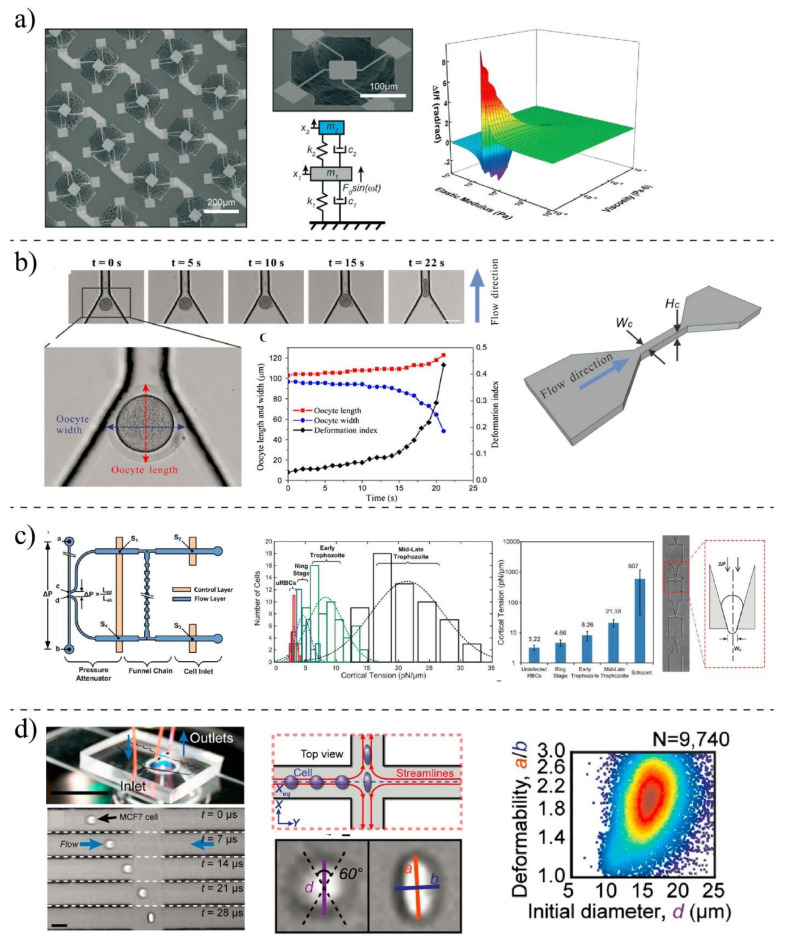
Different microfluidic and MEMS techniques for the mechanobiological characterization of cells: (**a**) Modeling cells as a two-degrees-of-freedom system and measuring their viscoelastic properties using a MEMS resonator. Reprinted with permission from [115]. Copyright Royal Society of Chemistry 2015. (**b**) Constriction channels to induce mechanical deformation onto oocyte cells and measuring their deformations as they pass through the tight channel. Adapted with permission from [135]. Copyright Springer Nature 2015. (**c**) A micro-aspiration integrated into a constriction channel for quantifying the deformability properties of cells by measuring the threshold pressures. Reprinted with permission from [136]. Copyright Royal Society of Chemistry 2012. (**d**) Hydrodynamic stretching of cells and high-throughput assay to measure the index of cells and investigate the deformability of cells. Reprinted with permission from [102]. Copyright (2012) National Academy of Sciences.

Researchers at MIT University developed a suspended MEMS resonator [101] to characterize the mechanobiological properties of ≈10^5^ single cells per h by integrating a constriction channel to the device at the apex of a micro-cantilever. By measuring the velocity and transit time of cells passing through the constriction channel, they evaluated the stiffness and friction of the cells. In another study, a MEMS resonator was proposed by Corbin et al. [115] to quantify the mechanobiological properties of human breast cancer cells (Figure 6a). They modeled the MEMS platform and the cells as a two-degrees-of-freedom system to estimate the mechanobiological properties of cells through the vibrational behavior of the microsystem. Then, they studied the shift resonant frequency of the system after and before chemically fixing the adherent cells to the resonating platform to predict their viscosity and elasticity. MEMS systems offer automated and rapid measurements; however, for mechanobiological measurement, they suffer from non-transparency and high stiffness compared to living cells [131].

In contrary to MEMS (normally made of silicon) systems, polymer-based microsystems offer more advantages. The mechanical properties of cells are closer to the mechanical properties of these polymers, so their in vivo microenvironment can be mimicked better. Due to the optical transparency of the polymer, the behavior of living cells and their deformations can be monitored with light microscopy at the same time [103,132]. With the aid of microfluidic devices, fast mechanobiological assays can be performed using reduced quantities of samples. Microfluidic techniques can be classified based on the mechanical stimuli used to deform the cells. Monitoring the cell movement as it passes through a constriction channel is one of the most straightforward techniques for studying the mechanobiological properties of living cells (Figure 6b). Under a hydraulic pressure difference, target cells are squeezed by the wall of the channel, which is marginally smaller than the diameter of the cell. With the aid of the constriction channel, various parameters such as entry time, passage time, elongation, and recovery time can be quantified. Clogging and channel blockage are the main limitations of these devices [133,134].

Deformation can be made with the aspiration technique in which the concept of conventional MA is mimicked to measure the mechanobiological properties of the cell (Figure 6c). A cell is partially aspirated into a microfluidic channel and deformed through a series of funnel-shaped constrictions. Meanwhile, the elongation of the cell is measured by a microscope and camera to infer the rheological properties of living cells [24,136]. Living cells can also be exposed to the hydrodynamic forces and deformation by designing microchannels in which various fluid stress stimuli are generated [108] (Figure 6d). In contrast to the mechanical confinement-induced deformation, cells can be deformed by shear stress within microchannels with a larger diameter than the cell’s diameter. The deformation index (DI) or stretch ratio is defined as the ratio of both axes of the cross-sectional area of the deformed cell and can be quantified by high-speed imaging. Using a high-speed imaging camera is one of the limitations of microfluidic-based fluid-induced deformation [101,114]. The optical stretcher is a popular method for the mechanobiological characterization of the suspended cells. This technique could be used to trap and stretch single cells based on the laser-induced momentum transfer. The stretching forces can be affected by the size, type of cells, refractive index, and laser power. Although optical stretching can measure the mechanobiological properties of cells, the imposing forces are not large enough to promote significant deformability to simulate *in vivo* conditions encountered by migrating cancer cells. Furthermore, the effects of the laser beam on the mechanobiological properties of cells are unknown and need further studies [99,130]. Electrical fields also can be implemented for the mechanobiological characterization of cells [22,113]. Whenever a single cell experiences an externally applied electrical field, it is swelled or expanded in size, which is a phenomenon known as electroporation. The electrical field increased the conductivity and permeability of the cell plasma membrane. The influx of small molecules through the open pores in the cell membrane causes the swelling and expansion of cells. Swelling ratios (before and after establishing voltage) of cells can be recorded to evaluate the deformability of cells.

### 3.3. Impacts of Nanoparticles on Structural Elements and Morphology of Cells

Any changes in the cytoskeletal structure of cells could lead to the alterations of the mechanobiological properties of cells. In order to understand the effects of NPs on the mechanobiological properties of cells, we need to study the physiochemical interactions between NPs and the three main filamentous proteins: intermediate filament, actin filament, and MT. In some studies, the disruption of subcellular structures has been reported due to the NPs uptake; however, the consequences of those changes to fundamental biological processes have been less investigated. The cytoskeleton is responsible for the basic functions of cells: (a) to preserve the morphology of cells, (b) to anchor organelles, (c) to physically connect cells to the microenvironment, (d) to produce internal forces for cells movement, (e) to help cells for division, and (f) endocytosis [81]. Therefore, any changes in the cytoskeleton organization could induce cellular dysfunction (see Table 2).

Most NPs are thought to penetrate cells through forming vesicles, and these membrane-bound vesicles transport NPs along MT to intracellular compartments. During this process, the NPs might have indirect interactions with cytoskeletal proteins and change their organizations. It is not clear how they interact with those proteins while they are encapsulated inside lysosomes and endosomes [17]. However, there are some evidence showing that NPs could directly interact with the cytoskeletal proteins. It has been found that carbon nanomaterials enter cells by adhesive interaction, enabling them to freely swim in the cytoplasm and directly interact with the subcellular structures of cells. For example, Lundqvist et al. [59] found the presence of MT in the protein corona formed around the SiO2 NPs, suggesting the direct NPs–proteins interactions. Direct or indirect interaction with NPs may negatively affect the biological functions [137,138,139]. Tian et al. [140] showed that single-wall carbon nanotubes could enter cells and alter cell morphology by disturbing the actin networks. They observed that these NPs cause an irregular actin network in comparison to untreated cells. Various NPs-related parameters such as the shape, size, surface chemistry, concentration, and incubation time are important in assessing the toxicity of nanomaterials in cytoskeleton. The shape of the NPs can induce different effects on the cytoskeletal structure of cells. It has been shown that unlike silica NPs with small aspect ratios, silica nanorods with large aspect ratios can largely change the organization of the actin filament, particularly in the vicinity of the cell membrane, resulting in serious damages to the cytoskeletal structures [141,142,143]. Ibrahim et al. [144] used different techniques such as SEM, TEM, and immunofluorescence analysis to study the cytoskeletal changes in osteoblast-like cells underexposure of titanium-based orthopedic and dental implants NPs (nano-Tio_2_). Smaller particles were found to be more disruptive to the actin and microtubule cytoskeletal network in comparison to larger particles. In another work, Holt et al. [145] used fluorescence lifetime microscopy to study the interactions of single-wall carbon nanotubes with HeLa cells. They showed that nanotubes preferentially interact with F-actin compared to G-actin and dramatically change their distribution. NPs even could disrupt the MT and actin network at non-toxic concentrations. Liu et al. [146] showed that bare gold NPs with the size of 20 nm alter the microfilament arrangement of endothelial cells more than NPs with the size of 5 nm. In this study, five types of gold NPs with different sizes and surface coatings were used to determine the viability and cytoskeletal change of endothelial cells. They found that gold NPs do not affect the viability of cells; however, the force balance between intracellular tension and paracellular forces is broken in 20 nm bare gold NPs-treated cells. In another study, the sub-lethal concentration of silver NPs was used to investigate cytoskeletal changes in neural cells [147]. They found that the percentage of AgNP-treated cells containing inclusions is doubled compared to control cells, indicating a significant disruption of actin filaments.

**Figure 7:**
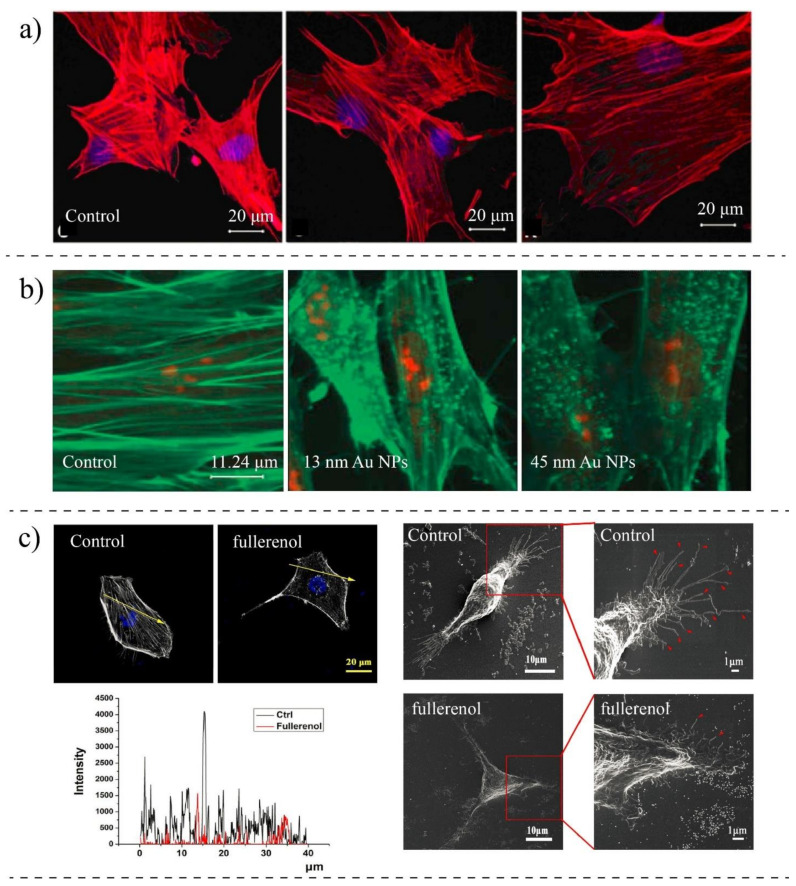
(**a**) Mesenchymal stem cells treated with silica (Si) and silica–boron (SiB): F-actin detected with red TRITC-phalloidin staining, and DNA stained with blue DAPI. Actins in control cells are packed longitudinally, while they are arranged transversally in treated cells [148]. Copyright 2014, Open Access Springer Journals. (**b**) Fluorescent imaging of human dermal fibroblasts stained for F-actin after three days exposure to gold NPs. F-actins appeared to be in dotted format compared to control cells. Reprinted with permission from [37]. Copyright Informa UK Ltd. 2010. (**c**) SEM images of MDA-MB-231 cells treated with fullerenol NPs compared to control cells. Treated cells show shorter protrusions in comparison to control cells, and the concentration of actin fibers has reduced after the uptake of NPs [151]. Copyright 2019, Open Access, Journal of Nanobiotechnology.

In vitro alternations in MT and F-actin concentrations and cytoskeletal destabilization have also been observed in cells, particularly under high concentrations of NPs. For example, Ogneva et al. [148] showed reduced F-actin content in silicon-treated mesenchymal stem cells compared to control cells (Figure 7a). Pisanic et al. [149] studied the effects of NPs concentrations on neuron cells. They found that by increasing the concentration of metal oxide NPs, the density of actin filaments is reduced, preventing them from getting mature under the stimulation of nerve growth factors. Mironava et al. [37] revealed that the cellular uptake of gold NPs disrupts actin fibers of human dermal fibroblast cells, and in contrast to the extended actin in control cells, in treated cells, actin filaments are broken and appeared as dotes (Figure 7b). However, no significant changes were found in actin or beta-tubulin protein levels. Choudhury et al. [150] studied the binding of nanosphere gold NPs to MT in the cell-free systems as well as in human lung carcinoma cells (A549) using Raman measurement, Fourier transform infrared (FTIR), and other imaging techniques. Their findings showed that gold NPs depending on their size and concentration might inhibit the polarization of MT. They also observed that MT networks are damaged and shrunken upon interaction with gold NPs compared to control cells.

NPs might have different affinities to different subcellular structures depending on their physicochemical properties [152,153]. Wen et al. [154] found that silver NPs tend to bind actin rather than tubules under electrostatic interactions. They used imaging techniques to visualize the organization of actin and tubulin proteins after treating with silver NPs (size 30 nm). They observed that the secondary structures of actins and tubules are changed due to the interaction with NPs, and alpha-helices of both proteins are decreased while their beta-sheets are increased. NPs-induced cytoskeletal changes could also cause significant morphological changes [155,156]. Rasel et al. [157] observed morphological changes of osteoblast cells after treating with boron nitride NPs, while they do not have adverse effects on the viability and the metabolism of cells. Ali et al. [158] showed that gold nanorods could change the cytoskeletal structure of oral squamous cell carcinoma. They observed morphological changes in cytoskeleton protrusions (filopodia and lamellipodia) when incubating cells with integrin-targeted gold nanorods. Qin et al. [151] found that the NPs-treated breast cancer cells have reduced the number and length of filopodia compared to control cells, causing them to lose their adhesion to the extracellular matrix (Figure 7c). Patra et al. [159] observed that gold NPs damage the cytoskeletal structure and induce profound morphological changes in human carcinoma cells (A549). Subbiah et al. [160] studied the morphological changes of A549, NIH3T3, and HS-5, and they found that silver NPs may induce changes in the topography of cells lines and treated cells appeared more rounded than untreated cells. Morphological changes could be influenced by concentration or incubation time. Wu et al. [161] proved that the density of filamentous proteins is reduced by increasing the concentration and exposure time of gold NPs in human aortic endothelial cells, causing topographic changes in the cell surfaces. In another work, Pernodet et al. [162] found that citrate-gold NPs profoundly affect the cell morphology of human dermal fibroblasts when the concentrations and exposure time are increased. They observed that the density of actin filament decreases in the presence of NPs by extending the exposure time, showing that the actin fibers are depolarized due to the cellular uptake of NPs.

In summary, in order to study the toxicity of nanomaterial in cells, the interaction of nanomaterial with subcellular structures, particularly cytoskeleton, needs to be taken into account. Nanomaterials, even under low concentration due to direct and indirect interactions with filamentous networks of cells, could change the main cellular structure and lead to mechanobiological changes in cells.

**Table 2:** Cytoskeletal changes due to the NPs–protein interactions.

Author	Cell Type	NPs Type	Methods	Cytoskeleton Changes
Pernodet et al., 2007 [162]	CF-31 (human dermal fibroblast)	Gold NPs (13 nm)	TEM, Confocal Imaging, Migration Assay	Modification in actin networks; NPs impaired motility and adhesion
Pi et al., 2013 [163]	MCF-7 (breast cancer)	Selenium NPs	AFM, Confocal Microscopy	The organization of F-actin is changed, and they are aggregated; Actin concentration is reduced
Choudhury 2013 [150]	A549 (lung cancer)	Citrate-capped Gold NPs (20–60 nm)	Raman, FTIR, TEM, Darkfield Microscopy, UV-Visible Spectroscopy	Inhibiting the polarization of MT; MT structures are damaged, affecting the dynamic equilibrium
Qin et al., 2018 [151]	MDA-MB-231 (breast cancer)	Fullerenol NPs	SEM, Fluorescence Imaging, AFM, Scratch Assay	The concentration of actin is reduced, the migration speed is reduced, disturbing actin assembly
Hot et al., 2012 [145]	HeLa (cervical cancer)	Single-wall carbon nanotube (1 ± 0.3 nm)	Fluorescence Imaging Microscopy	NPs cause cells to have shorter F-actin; Traction force is reduced; NPs do not affect G-actin and myosin II
Huang et al., 2010 [141]	A375 (melanoma)	Silica NPs (MSNs)	TEM, Confocal Microscopy, Western Blot	The actin structure is disorganized and disrupted with NPs; Cell migration is reduced
Patra et al., 2007 [159]	A549 (lung cancer)	Gold NPs	Confocal Microscopy	The morphology is changed; Treated cells are rounded compared to non-treated
Pisanic et al., 2007 [149]	PC12M (brain)	Fe_2_O_3_ NPs	TEM, Western Blot, Fluorescent Microscopy	Reduction in the formation of actin microfilaments; They are less organized; NPs diminish the ability for differentiation
Wu et al., 2012 [161]	HAEC (aortic endothelial cells)	Diesel exhaust particles (DEPs)	AFM, Fluorescent Imaging	Cells became degraded; Cellular cytoskeletal structures were impaired
Wen et al., 2013 [154]	Acting and tubulin proteins (cell-free system)	Silver NPs	TEM, Hyperspectral Imaging,	Inducing changes in the secondary structures; Silver NPs tend to bind actin vs. tubulin
Cooper et al., 2015 [147]	B35 (neuroblastoma)	Silver NPs	Immunocytochemistry	NPs induce F-actin inclusion, disrupting the actin function
Rasel et al., 2015 [157]	Osteoblast cells	Boron nitride NPs	AFM, TEM, X-Ray	They do not affect the morphology of cells
Liu et al., 2017 [146]	HUVEC (Endothelial cells)	Gold NPs-coated with PEG (20 nm)	Fluorescent Microscopy, Traction Force Microscopy	NPs re-arranged actin filaments; Inhibition of Rock activity reduced the polymerization of actin; Reducing the focal adhesion
Vieira et al., 2017 [164]	CCD1072Sk (Normal cells-skin)	Gold NPs and silver NPs	Immunofluorescence Imaging, Cytofluorometry	NPs impair the F-actin;Cytoskeletal reorganization; Cells lose the cell polarization (without losing their viability)
Ali et al., 2017 [158]	HSC-3 (tongue cancer)	Gold nanorods coated with PEG and REG	Western Blot, DIC Microscopy, Scratch Assay	The cytoskeletal proteins are rearranged; Cytoskeletal protrusions (filopodia and lamellipoda) are reduced
Beaudet et al., 2017 [48]	HeLa (cervical cancer)	AuNPs, Swarna Bhasma	Fluorescent Imaging	Larger particles disrupted the microtubules networks
Ibrahim et al., 2018 [144]	SaOS-2 (bone cancer)	TiO_2_ spherical NPs	Hyperspectral Imaging, Fluorescent Imaging, Western Blot	The actin and microtubule cytoskeletal networks are disorganized
Kralovec et al., 2020 [143]	A549 (lung cancer)	Fe_3_O_4_@SiO_2_	Fluorescent Imaging, Western Blot	Severe disruption of the actin filament and microtubules
Kota et al., 2021 [165]	VSMCs (vascular smooth muscle cells)	ZIF-8 NPs	AFM, Fluorescent Imaging, Polymerization Assay	Morphological changes and cytoskeletal disorganization were observed; NPs caused changes in actin filaments at basal and apical surfaces.

### 3.4. Impacts of NPs on Cell Stiffness

The resistance of cells to the external forces can provide information regarding the health state of cells. The resistance of cells to the applied forces can be expressed by stiffness. This parameter shows the relationship between the stress and the applied strain and can be characterized by the Young’s modulus (E) of cells (unit in Pascals). Multiple studies have shown that cells express different elasticities, depending on their diseased state [76,156]. In some diseases such as malaria [133], the level of stiffness may be increased. However, for other diseases such as cancer, several studies are showing that the stiffness of cancer cells is reduced compared to their normal counterparts [75,166,167]. In healthy cells, the cytoskeleton is well organized, and the density of actin filaments, the main constituent of the cytoskeleton system, is higher, enabling cells to resist external forces. These organized structures cannot be observed in malignant cells, and the density of filamentous proteins (stress fibers) is lowered, leading to a softer cellular structure [168]. The relationship between the cytoskeletal mechanical properties and the biological function of healthy and cancerous cells can provide a meaningful approach to evaluate the health state of cells [95]. Therefore, modulating the mechanical properties at the cellular level could suggest an approach for cancer treatment. This could happen by targeting cytoskeletal filaments that play significant roles in the mechanical properties. For this purpose, many anti-cancer drugs are designed to target the cytoskeletal structure and induce cytotoxicity and inhibit metastasis. For example, Taxol suppresses the depolymerization of tubulins and inhibits metastasis [21]. Rotsch et al. [169] showed that the pharmacological targeting of actin fibers could significantly affect cell mechanics. Mechanobiological measurements of cells could provide a platform to assess the effectiveness of anti-cancer drugs delivered through NPs or NPs alone. As discussed earlier, NBI could disorganize the cellular structure of cells and impose changes in the mechanobiology of cells. These mechanobiological changes could be measured using methods introduced in the previous section. AFM is the most widely used technique for cell stiffness due to the high-resolution imaging and quantitative measurements. Many researchers have used this tool to investigate the effect of NBI on cell stiffness (Table 3).

**Figure 8:**
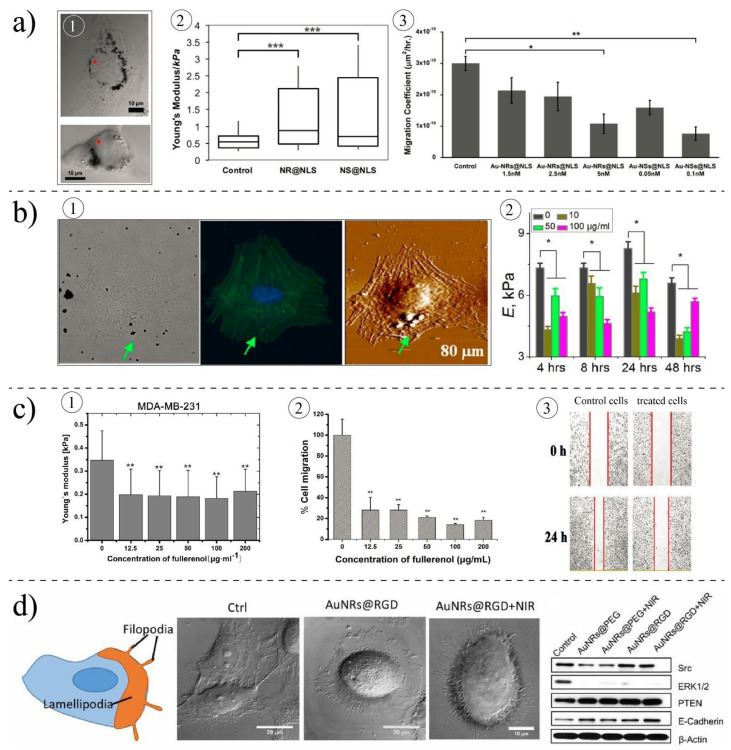
Effects of NPs on cell mechanics. (**a**) Effects of gold nanospheres (NS) and nanorods (NR) on HEYA8 cell, (**a1**) TEM images of cells treated with NPs, (**a2**) treated cells are become stiffer compared to control cells, (**a3**) effects of NPs on the migration ability of cells. Reprinted with permission from [172]. Copyright American Chemical Society 2017. (**b**) Effects of DEPs on HAECs cells, (**b1**) bright field, fluorescent, and AFM images of cells treated with NPs and their distribution in cells, (**b2**) elasticity (E) of treated cells compared to control cells after different exposure times (* *p* < 0.01) [161]. Copyright 2012, Open Access, PloS ONE Journal. (**c**) Effects of small fullerenol on human breast cancer cells, (**c1**) elasticity of MDA-MB-231 cells is reduced upon interaction with NPs, (**c2**) migration ability of cells is reduced, (**c3**) scratch assay measurement for treated cells and control cells [151]. Copyright 2018, Open Access, Nanobiotechnology Journal. (**d**) Effects of gold nanorods on HSC. NPs could change the morphology of cells and reduce the movement ability of cells by changing the protein expression. Reprinted with permission from [158]. Copyright (2017) National Academy of Sciences.

NPs may increase the stiffness of cells through interactions with the different components of cells, which play a central role in cell mechanics. Buyukhatipoglu et al. [170] used AFM to estimate the stiffness of porcine aortic endothelial cells (PAEC) following bare iron oxide NPs uptake. Their study showed that cell length increases after incubating PAEC with NPs, and actin stress fibers are stretched across the cell body, causing a significant increase in the stiffness of endothelial cells. Ogneva [148] showed that silica-based NPs might cause mesenchymal stem cells to have higher stiffness than control cells. The AFM measurements showed that the stiffness of the cells could be increased by 61% after interaction with NPs. They claimed that after NPs uptake, the F-actin content is reduced, and their structures are reorganized, altering their cell mechanics. Subbiah et al. [160] studied the effects of different hybrid NPs on different cells lines: A549, NIH3T3, and HS-5 using AFM measurements. The results revealed that after treating cells with NPs, their mechanical properties are increased. Pietuch et al. [171] revealed that gold nanosphere-treated MDCK II cells display a concentration-dependent stiffness. With AFM measurements, they found that a higher concentration of CTAB-coated nanospheres gold (>3 µg/mL) can significantly increase the stiffness of cells, whereas the low concentration (0.5 µg/mL) reduces the cell stiffness compared to non-treated cells. Ali et al. [172] carried out a study on the nucleus stiffness of ovarian cancer cells (HEY A8), and they proved that gold nanorods designed to target the cell nucleus could increase the stiffness of the cells, slow down the migration of cancer cells, and suppress the invasive ability of cells (Figure 8a). They observed that gold nanorods uptake enhances the expression of actin inner nuclear membrane lamin A/C protein.

On the other hand, some other works show the opposite results; the stiffness of cells is reduced due to the internalization of NPs in the cells. Pi et al. [163] observed that the internalization of selenium NPs could remarkably decrease the Young’s modulus of MCF-7 cells. Selenium NPs induced changes in the organization and regulation of cytoskeletal structures of cells by disrupting F-actin. Babhosseini et al. [173] designed a microfluidic device integrated with multiple constriction channels to study the mechanobiological changes due to the internalization of poly lactic-co-glycolic acid (PLGA) NPs coated with an anti-cancer drug (sphingosine kinase inhibitor). They observed that treated cells could pass through the constriction channel faster than the control cells, indicating a reduction in the stiffness of cells. It was also observed that the concentration of actin filament and stiffness are increased when NPs alone loaded to cells. The findings of Wu et al. [161] revealed a gradual down-regulation of the cytoskeletal component of Human Aortic Endothelial Cells (HAEC) cells after exposure to diesel exhaust particles, causing them to have a softer body than non-treated cells (Figure 8b). Qin et al. [151] investigated the biophysical changes in human breast cancer after interaction with small fullerenol NPs (Figure 8c). Their results revealed that fullerenol NPs disorder the arrangement of actin fibers and cause them to become thinner compared to the straight, strong, and well-arranged fibers in control cells. The Western blot measurement also confirmed a strong reduction in the content of F-actin and G-actin. The effects of those changes were also observed in the Young’s modulus of cells. Their AFM measurements showed a significant decrease in the Young’s modulus of MDA-MB-231 and MCF-10A due to the internalization of fullerenol NPs.

**Table 3:** Effects of various NPs on the mechanobiological properties (stiffness) of cells.

Author	Cell Type	Nanoparticles	Parameter	Techniques	Results
Buyukhatipoglu et al., 2010 [170]	PAEC (endothelial cells)	Iron oxide	Young’s modulus	AFM	The stiffness is increased
Yangzhe Wu et al., 2012 [161]	HAEC (endothelial cells)	Diesel exhaust particle (DEP)	Young’s modulus	AFM	Young’s modulus is reduced depending on the doses
Jinag Pi et al., 2012 [163]	MCF-7 (breast cancer)	Selenium NPs	Young’s modulus	AFM	The Young’s modulus is reduced. Adhesion is reduced
Subbiah et al., 2013 [160]	A549 (lung cancer), NIH3T3 (fibroblasts) HS-5, (fibroblasts)	Hybrid NPs (Silver NPs and single-walled carbon nanotube)	Young’s modulus	AFM	Stiffness is increased
Ogneva et al., 2014 [148]	mesenchymal stem cells	Silica-based NPs	Young’s modulus	AFM	Stiffness is increased
Rasel et al., 2015 [157]	osteoblast cells	Boron nitride nanoparticle (BN NP)	Young’s modulus	AFM	Stiffness is increased
Anna Pietuch et la. 2015 [171]	MDCK II cells (kidney)	Gold nanorods and spherical NPs	Young’s modulus	AFM	Stiffness varies depending on the Au concentration
Babhosseini et al., 2015 [173]	MDA-MB-231(breast cancer)	SphKIs with NPs	Deformability-related parameters (passage time and velocity)	Microfluidic (Constriction channel)	Cells became softer (reduced stiffness)
Ali et al., 2017 [172]	HEY A8 (ovarian cancer)	Gold nanorods	Young’s modulus of nucleus	AFM	Stiffness is increased and NPs inhibited metastasis
Qin et al., 2018 [151]	MDA-MB-231 (breast cancer), MCF7(breast cancer)	Fullerenol NPs	Young’s modulus	AFM	Stiffness is decreased
Kashani et al., 2019 [174]	A549 (lung cancer)	Gold nanospheres/nanostars	Young’s modulus	AFM	Stiffness is decreased
Rasel et al., 2019 [175]	osteoblast cells	Boron nitride NPs	Young’s modulus	AFM	Stiffness is increased
Pastrana et al., 2019 [176]	NIH3T3 (fibroblasts)	Multiwall Carbon NPs	Young’s modulus	AFM	Stiffness is decreased
Wilhelm et al., 2021 [177]	F9 murine embryonal carcinoma cells	Magnetic NPs (Iron oxide NPs)	Young’s modulus	Parallel plate rheometer	Stiffness is increased

### 3.5. Impacts of Nanoparticles on Migratory Ability of Cells

The migration of cells plays a highly important role in wound healing and cancer metastasis. For migration, different steps are performed: (a) the cell body is polymerized to establish a front to the rear polarity axis, (b) protrusion of the cell membrane to form lamellipodia at the leading edge, and (Figure 8d), (c) cell body retraction. The cytoskeleton contributes to all these steps so that NP–cytoskeleton interactions could alter the mobility ability of cells. By decreasing the cell adhesion and altering the expression of cell migration-related proteins, NPs could change the migration potential of cells. By reducing the cell adhesion, cells cannot provide sufficient traction force to pull cells forward. The findings of Prenodet et al. [162] revealed that the density of F-actin in human dermal fibroblast is dramatically reduced, particularly in a layer adjacent to the substrate when incubating with citrate-capped gold NPs after six days, and consequently, cells showed less ability to migrate and proliferate. Hou et al. [178] showed that the adhesion of cells is reduced by treating with TiO2 NPs, slowing down the migration ability of cells. Zhou et al. [179] studied the effects of gold nanorods on three different cells lines: MDA-MB-231 (human breast cancer cells), PC3 (prostate cancer cells), and B16F10 (mouse melanoma cells). Their results revealed that rod-shaped gold NPs could effectively inhibit the migration and invasiveness of cells. Their investigations showed that NPs, once they are in cells, can down-regulate the expression of energy-related proteins. They showed that ATP production is reduced and subsequently inhibits the assembly of F-actin, which is important for cell migration. In contrary to non-treated cells, they showed that NP-treated cells no longer have stressed F-actin. NPs may alter the migratory ability of cells without inducing cytotoxicity. Vieira et al. found [164] that silver and gold NPs degrade the concentration and F-actin, consequently impacting the migratory ability of cells. Their results revealed no change in the viability of cells due to the interaction with silver and gold NPs; however, NPs reorganized actins and subsequently decreased the migration. In another work, Pan et al. [180] showed that gold NPs (15 nm) could inhibit the endothelial growth factor of HUVEC cells. Their wound-healing assays revealed that the cell migration and tube forming are reduced after incubating with gold NPs. NPs may be used to manage the metastasis ability of invasive cancer. Effects of the small size of fullnernol NPs [151] were examined on the metastasis behavior of invasive (MCF7) and highly invasive (MDA-MB-231) human breast cancer cells. By staining the actin filaments, immunofluorescence imaging, and Western blot analysis, the authors confirmed that the actin concentration is altered, and the cytoskeleton assembly is disrupted. It was also found that cytoskeleton reorganization alters the intracellular distribution of integrin, causing cells to lose their adhesion ability. They concluded that fullerenol NPs are able to significantly inhibit the migration of malignant cells when the concentration of NPs is increased. Ali et al. [158] showed that the uptake of NPs reduces the migration ability of HSC cells by altering the concentration of migration-related proteins, suggesting the potential application of nanorods for controlling cancer metastasis. Chan et al. [181] showed that gold NPs with small size (3–5 nm) could inhibit the migratory potential of RF/6A cells while inducing no change in the viability and adhesion of cells.

There are also few studies showing the opposing effects of NPs on cell migration; migration is increased by NBI. Liu et al. [182] observed an increase in the migration of human lung cancer cells treated with small gold NPs (10 nm). Their results showed that gold NPs could notably facilitate the invasion of 95D cells. The enhanced migration activity could be associated with increased expression of metalloproteinase 9. Shahhosseini et al. [183] observed the contradictory effects of gold NPs on the migration of human colon adenocarcinoma, melanoma, and their nano-cancerous counterparts. They found that gold NPs reduce the migration of tumor cells by 20% while enhancing the migration of non-cancerous cells by 13%. NPs could cause non-migratory cells to migrate and develop diseases. For example, unlike non-migratory VSMSc cells, zeolitic imidazolate framework-8 (ZIF-8) NP-treated cells migrate and could cause cardiovascular disease [165]. The mechanical changes (Young’s modulus) in cells could influence their migratory abilities. Both the increase and decrease in migratory ability of cells could be influenced by the stiffness of cells [25]. As earlier discussed, NBI (or some anti-cancer drugs) could disorganize the cytoskeleton and cause both an increase and decrease in stiffness of cells, so stiffness measurements could potentially be used as a platform to evaluate NBI for nanomedicines design and predict the mobility ability of cells.

## 4. Summary, Conclusions and Outlooks

Cancer treatment and diagnosis with the help of nanotechnology is an interdisciplinary field focused on biology, chemistry, engineering, medicine, and physics. Therefore, it is necessary to consider various aspects of NBI to design safe and effective therapeutic and diagnostic NPs-based systems. Although studying cellular uptake, toxicity, and intracellular localization is an essential step in designing nanomedicines [156,184,185,186], they may not alone cover all aspects of NBI and be sufficient to design safe and efficient nanomedicines. The cellular uptake of NPs might cause different kinds of changes to cellular functions even at very low concentrations. NPs at sub-toxic concentrations might influence cell mechanics and alter the proliferation, differentiation, or migration of cells. Due to the nanosize and unique physiochemical properties, NPs are expected to interact with different compartments of cells directly or indirectly, such as the cell membrane, cytoskeleton, and organelle, and those interactions might alter cellular functions and structures. The effects of these changes could be reflected in the mechanobiological properties of cells. Therefore, monitoring the mechanobiological of treated cells could potentially provide a platform to indirectly study the NBI and be used to improve the efficacy of NP-based systems. Mechanobiological changes have been reported in various types of cells after incubation with NPs. However, the exact effects of NPs on cell mechanics are unknown and have been investigated only by a few studies. So, we believe there is an urgent need to thoroughly study the impacts of NPs on cell mechanics in the NBI field, particularly at sub-lethal concentrations. Many challenges have not yet been addressed. For instance, it is unclear how the physicochemical properties of NPs influence the mechanobiology, to what extent NPs could alter the mechanobiological properties of cancer cells, and most importantly, how those changes benefit and risk treatment.

Further progress in this field will help develop the application of intracellular NPs to regulate cell mechanics for cancer treatment. Many studies have confirmed mechanobiological changes and increased migration/invasiveness in cells during cancer [25]. On the other hand, NPs were shown to be effective in the mechanobiological modification; hence, NPs could potentially be used to alter the mechanobiology of cells and improve the treatment and management of cells; however, more detailed studies need to be done to explore their positive or negative roles in cancer progression. In addition to investigating the uptake, biocompatibility, and localization of different NPs in cancer cells, studying the mechanobiological properties of treated cells is suggested to improve NPs-based treatments. Understanding the biocompatibility of NPs and their influence on the mechanobiology of cancers cells takes us one step closer to optimizing nanomedicines for safe and effective cancer treatment.

## Figures and Tables

**Table 1:** Different techniques for mechanobiological measurements of cells.

Techniques		Cell Type	Mechanical Stimuli	Important Parameters	Advantages	Limitations
**Classical Techniques**	Atomic Force Microscopy (AFM)	MCF7 [104]; Human bladder [96]	Cantilever micro indention	Tip deflection, Young’s modulus	High-resolution measurement; Provids both structural and mechanical information for local, whole, and interior measurements [23,97]	Low throughput; Mechanical hitting of AFM tip may affect cell activities and position of probe; Requires a high-resolution microscope
	Micropipette aspiration (MA)	Human cartilage [98]; Colon cancer cells [105]	Negative force	Young’s modulus	Low-cost and well-established method	Limited spatial resolution; Low throughput; For suspended cells only
	Magnetic twisting cytometry (MTC)	Melanoma [100]; MCF7 [106]	Force is applied by magnetic beads	Stiffness and Young’s modulus	Inducing little heat and photodamages compared to optical tweezer [10]	Resolution limitation; Inducing non-uniform stress; Beads are localized randomly on cell; Attachment angle affects the displacement
	Optical tweezers (OP)	RBC [99,107]	Laser-induced surface force	Deformation index	Without physical contact	Only for suspended cells; Damaging consequence of optical heating on cells; Limited magnitude of forces
	Parallel plate	Epithelial ovarian cancer [23]; MCF7 [106,108]	Shear stress	Aspect ratio	Homogeneity of the applied shear stress; Simplicity; Ability to study cell population	Need bulky devices; Large amount of reagents; Difficult to visualize deformation
**Microfluidic Techniques**	Fluid-induced deformation	PBMCs [102]	Fluid shear stress	Deformation index, size	High throughput; Simultaneously, other chemical assays can be done; The measurment can be done continuously; Contactless deformation; Applicable for both suspended and adhered cells	Needing expensive high-speed camera for imaging
	Constriction-induced deformation	K562 [109]; MDA-MB-231 [110]	Mechanical squeezing	Passage time, entry times, stiffness	Wide-ranging applications in cell deformation; Applicable for different geometry structures; Adjustable dimension for different cell types	Clogging and channel blockage; Possible effects of friction between cell and channel’s wall on measurements; Ignoring the effects of membrane rigidity and viscosity
	Aspiration-induced deformation	Neutrophils [24]	Negative pressure	Young’s modulus, cortical tension	Straightforward method; Well-established mathematical model	Leaking problem; Rectangle-like cross-section of microfluidic channels; Time-consuming process; Requiring high-vacuum pressure
	Optical stretcher	MCF7 [106]; MCF-7, MCF-10, MDA-MB-231 [111]; Red blood cells [99]; Melanoma cells [112]	Optically-induced surface forces	Deformation index, cell elasticity	No physical contact; Relatively high-throughput measurements	Alignment problem; Optical heating; Thermal damage
	Electrical-induced deformation	MCF-10A, MCF-7 [113]	Electroporation-induced swelling	Deformation index, size of cells	Fast heat dissipation; Better resolution; Automation and parallelization of test with reduced amount of samples	High energy consumption and high voltage
**MEMS Techniques**	Suspended microcantilever	Circulating tumor cells [114]; Fibroblast [101]	External actuator	Frequency of cantilever, passage time, transit time	All-inclusive systems; Parallel analysis; Better quality factor; Automation	Fabrication is expensive; Non-transparent channels; High stiffness of silicon; calibration process
	MEMS resonator	MCF7 [115]	External actuator	Frequency of cantilever	High throughput	Expensive fabrication; Requiring external electrical system; Only for adherent cells

## Abbreviations

AFM	Atomic force microscopy
AgNPs	Silver nanoparticles
CTAB	Cetrimonium bromide
DEPs	Diesel exhaust particles (DEPs),
FTIR	Fourier transform-infrared
HAECs	Human aortic endothelial cells
HSC	Human oral squamous cell carcinoma
HUVEC	Human umbilical vein endothelial cells
MEMS	Micro-electromechanical systems
MP	Micropipette
MT	Microtubules
MTC	Magnetic twisting cytometry
NBI	Nano-bio-interaction
NPs	Nanoparticles
NRs	Nanorods
NSs	Nanospheres
OP	Optical tweezer
PAECs	Porcine aortic endothelial cells
PLGA	Poly lactic-co-glycolic acid
SEM	Scanning electron microscopy
TEM	Transmission electron microscopy
VSMCs	Vascular smooth muscle cells
